# Editorial: Gender pressure in cancer: susceptibility, progression and treatment of the neoplastic disease

**DOI:** 10.3389/fendo.2023.1270199

**Published:** 2023-09-04

**Authors:** Marina Bacci, Teresa Gagliano

**Affiliations:** ^1^ Department of Experimental and Clinical Biomedical Sciences, University of Florence, Florence, Italy; ^2^ Department of Medicine, University of Udine, Udine, Italy

**Keywords:** cancer, gender, sex hormones, therapy response, predictive biomarkers

Gender impact on cancer susceptibility has emerged as one of the most reliable discoveries in cancer research.

The original articles presented in this Research Topic show that gender can play a crucial role in the development and prognosis of different types of diseases, including cancers. Increasingly, evidence has revealed that genetics and sex hormones can affect tumorigenesis and anticancer therapy response, influencing tumor progression. The aim of this Research Topic was to provide an updated overview on the role of gender in tumor etiology and progression in order to summarize current knowledge and state of the art.

The study published in 2022 by Biagetti et al. aimed to identify predictive factors of response to therapy based on somatostatin receptor ligands (SRL) in patients affected by acromegaly. The authors have developed a predictive model that considers different variables, such as age at diagnosis, gender, basal level of growth hormone, and diameter of tumor, and formulated a new prognostic score for clinical application. In particular, they showed that the female gender together with the levels of gender hormone are associated with a better response to SRL.

In addition to the genetics that obviously differs between male and female, sex hormones could also have an important impact on tumor progression. An example of this has been demonstrated by Fu et al., who investigated the clinical characteristics of patients simultaneously affected by breast and thyroid cancer to identify possible molecular mechanisms of tumorigenesis. Interestingly, differentiated thyroid carcinoma patients are characterized by high levels of estrogen receptor, a condition that characterizes more than 70% of breast cancer patients. Through statistical analysis and bioinformatics approaches, the authors show that co-occurrence risk of breast and thyroid cancer in the same individual is higher than in the general population and double primary cancers share a common pathogenic mechanism. In particular, these patients are characterized by the overexpression of COMP, which in turn could promote oncogenesis and progression of tumors through estrogen signaling and may increase the risk of thyroid cancer in breast cancer patients or breast cancer in thyroid cancer patients.

The work by Zhang et al. described a method to predict overall survival in mesothelioma patients in a Chinese population. The authors discovered that gender histology was strictly related to survival rate. This data highlight once again that gender should be taken into consideration when a patient is diagnosed with cancer, as this could have an impact not only on overall survival but also on treatment response.

Zhou et al. have demonstrated that patients with pancreatic signet ring cell carcinoma are more likely to be male, compared with pancreatic duct adenocarcinoma that is diagnosed more often in females. Once again, the authors underline a different distribution of diagnosed neoplasia based on gender.

All the manuscripts published in our Research Topic underline how cancer susceptibly is dramatically affected by gender, and this should become an important parameter during patient management.

## Author contributions

MB: Writing – original draft. TG: Writing – review & editing.

